# Nitrogen-Doped Carbon Nanotube-Supported Pd Catalyst for Improved Electrocatalytic Performance toward Ethanol Electrooxidation

**DOI:** 10.1007/s40820-017-0129-5

**Published:** 2017-02-14

**Authors:** Ying Wei, Xinyuan Zhang, Zhiyong Luo, Dian Tang, Changxin Chen, Teng Zhang, Zailai Xie

**Affiliations:** 1 College of Materials Science and Engineering, Fuzhou University, Fuzhou, 350108 Fujian People’s Republic of China; 2 College of Chemistry, Fuzhou University, Fuzhou, 350108 Fujian People’s Republic of China; 3 Department of Micro/Nano Electronics, School of Electronic Information and Electrical Engineering, Shanghai Jiao Tong University, Shanghai, 200240 People’s Republic of China

**Keywords:** Direct alcohol fuel cells, Hydrothermal carbonization, Nitrogen-doped carbon nanotubes, Pd-based catalyst, Ethanol electrocatalyst

## Abstract

In this study, hydrothermal carbonization (HTC) was applied for surface functionalization of carbon nanotubes (CNTs) in the presence of glucose and urea. The HTC process allowed the deposition of thin nitrogen-doped carbon layers on the surface of the CNTs. By controlling the ratio of glucose to urea, nitrogen contents of up to 1.7 wt% were achieved. The nitrogen-doped carbon nanotube-supported Pd catalysts exhibited superior electrochemical activity for ethanol oxidation relative to the pristine CNTs. Importantly, a 1.5-fold increase in the specific activity was observed for the Pd/HTC-N1.67%CNTs relative to the catalyst without nitrogen doping (Pd/HTC-CNTs). Further experiments indicated that the introduction of nitrogen species on the surface of the CNTs improved the Pd(0) loading and increased the binding energy.

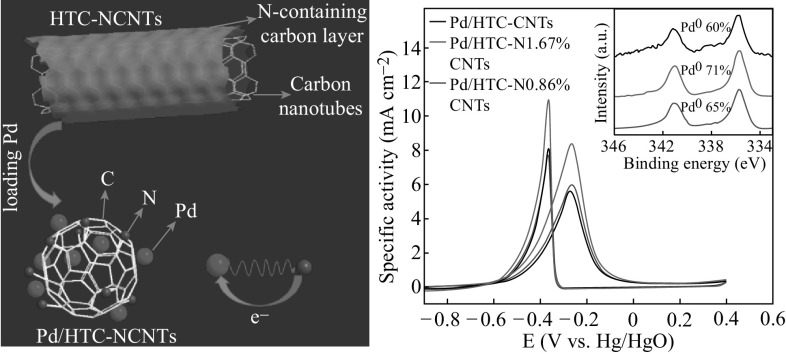

## Highlights

- Hydrothermal carbonization (HTC) enabled the deposition of an N-doped carbon layer on the surface of carbon nanotubes (CNTs).
- Nitrogen-doped CNTs facilitated the uniform distribution of Pd nanoparticles.
- The interaction between nitrogen in the CNTs and Pd favored the existence of metallic Pd in the catalysts.
- Pd/HTC-N1.67%CNTs showed the highest specific activity toward ethanol oxidation.

## Introduction

Direct alcohol fuel cells (DAFCs) have attracted much attention recently due to their high efficiency for energy conversion as well as their low environmental impact [1–3]. Among small organic molecules such as methanol, ethanol, and ethylene that yield energy upon oxidation, ethanol is the most ideal fuel because of its abundant reserves, low toxicity, and facile storage and transport. For commercial application, anode catalysts with high activity and stability, such as the widely accepted Pt-based catalysts, are critical for high-performance DAFCs [4, 5]. To address the issues of the high price and low reserves of Pt-based catalysts, many studies have been devoted to developing Pd-based catalysts by regulating the active phases and adding various promoters and varying catalyst supports. For example, certain studies have focused on the addition of secondary elements such as Sn, Cu, Ni, and Au to carbon-supported Pd to improve the electrocatalytic activity and stability for ethanol oxidation due to the bimetallic synergetic effect [6–8]. Moreover, trimetallic counterparts were also found to exhibit greatly improved catalytic performance and provide greater functionality [9–11].

The support materials also play a significant role in the catalytic reaction. Carbon-based support materials such as active carbon, graphite, and carbon nanotubes (CNTs) have been widely investigated. Among these materials, CNTs have earned distinction based on their high surface area, high length to diameter ratio, and good electrical conductivity [12, 13]. In addition to the common carbon-class supports, some transition metallic carbides, nitrides, phosphides, and corresponding hybrid composites have been employed as efficient supporting materials that positively impact the catalyst activity [14–16]. Additionally, some studies have shown that doping CNTs with heteroatoms [17–22] is an effective way to tune their intrinsic properties. The substitute heteroatoms can provide more initial nucleation sites for the formation of noble metal nanoparticles and also enhance the interaction with the nanoparticles, thereby improving the electrocatalytic activity [18]. Nitrogen-doped carbon nanotubes (NCNTs) are particularly promising candidates owing to the strong electron donor behavior of nitrogen, which enhances the *π* bonding and the basic properties of the NCNTs [19]. A study by Chetty et al. [20] showed that PtRu nanoparticles supported on nitrogen-doped multiwalled carbon nanotubes exhibited higher activity for methanol oxidation than the same catalysts supported on undoped nanotubes. The introduction of nitrogen-containing functional groups onto a carbon support may improve the catalytic activity by influencing the particle nucleation and growth and changing the electronic structure of the catalyst, as well as increasing the chemical binding energy between the support and catalyst particles, thereby enhancing the durability [21–23].

Furthermore, the use of inexpensive, sustainable feedstocks to produce N-doped carbon materials conforms to the concept of green chemistry. Generally, NCNTs can be synthesized by two methods, i.e., post-doping treatment or direct synthesis [19, 24]. The post-doping treatment is a multi-step process requiring high-cost apparatus. However, the alternative in situ synthesis requires an expensive precursor, complicated equipment, and is environmentally unfriendly. Therefore, finding a more economic and green strategy to synthesize NCNTs is necessary [25].

The hydrothermal carbonization (HTC) technique is a sustainable approach for producing NCTNs by using inexpensive feedstocks as carbon precursors. Interestingly, Titiricic et al. [26] recently reported that the HTC process enables homogeneous coating of nitrogen-doped carbon layers on the surface of CNTs, leading to superior supercapacitor performance. In spite of the paucity of examples, it is expected that nitrogen functionalization of CNTs by the HTC method could afford interesting supports for anchoring Pd for heterogeneous reactions.

In this study, surface functionalization of CNTs is performed by the HTC method using glucose and urea. The HTC process allows the deposition of thin nitrogen-doped carbon layers on the surface of the CNTs. By controlling the ratio of glucose to urea, nitrogen contents of up to 1.7 wt% could be achieved.

## Experimental

The CNTs were functionalized by oxidation with concentrated HNO_3_ (70%) at 110 °C for 4 h. An appropriate amount of urea, glucose, and pretreated CNTs was mixed and subjected to hydrothermal treatment at 180 °C for 6 h. After washing and drying, the collected powder was calcined at 900 °C for 4 h under Ar atmosphere. The different supporting materials, i.e., without N-doping (HTC-CNTs), doped with 0.86%N (HTC-N0.86%CNTs) and 1.67%N (HTC-N1.67%CNTs), were obtained by similar procedures. Elemental analysis was performed by using an elemental analyzer (Vario EL Cube). The elemental analysis results for the different supporting materials are summarized in Table 1.

**Table 1:** Elemental analysis data for supporting materials

Supporting materials	Elemental compositions (wt%)			
	N	C	H	O
HTC-CNT	0	90.28	2.60	6.12
HTC-N1.67%CNT	1.67	88.60	2.64	6.09
HTC-N0.86%CNT	0.86	89.29	2.66	6.19

The Pd-based catalyst was synthesized using PdCl_2_ as the metal source, NaBH_4_ as the reducing agent, and the different composites as supporting materials. Thus, 48 mg of the pretreated HTC-CNTs and 1.3 mL of 18.9 mM H_2_PdCl_4_ were mixed with 30 mL of deionized water under ultrasonic stirring. The pH of the solution was adjusted to 9 by using 1 M NaOH. An appropriate amount of NaBH_4_ (Pd/NaBH_4_ = 1:8 mol%) was then added dropwise to the aforementioned solution (with a nominal Pd loading of 5 wt%), and the mixture was stirred vigorously for 4 h. The resulting precipitate was filtered and washed several times with ultrapure water before drying overnight at 60 °C. The weight percentage of Pd in the catalysts, determined by inductively coupled plasma–atomic emission spectroscopy (ICP–AES), was 4.9 ± 0.1 wt%.

X-ray diffraction (XRD, Rigaku D/max-IIIC) was performed using a copper *K*α source (*λ* = 1.5406 Å). The microstructure was analyzed using high-resolution transmission electron microscopy (HRTEM, Tecnai G2 F20 S-TWIN) at 200 kV. X-ray photoelectron spectroscopy (XPS, ESCALAB 250, Thermo Scientific, Inc.) was performed using a monochromatic Al *K*α source at 10 mA and 15 kV. The electrochemical measurements were conducted by using a CHI660D electrochemical workstation (Chenhua Inc., Shanghai, China) in a conventional, sealed, three-electrode system. Raman spectra were collected between 500 and 3100 cm^−1^. The light source was a 532 nm argon laser, and the data were collected with 50-s exposure.

A Pt wire and a Hg/HgO electrode were used as the counter electrode and reference electrode, respectively. A glassy carbon electrode (GCE, Ø3 mm) was employed as the working electrode; the working electrode was prepared as follows: 5 mg of the catalyst was ultrasonically mixed with 25 µL of 20 wt% Nafion solution and 975 µL of isopropyl alcohol to prepare a homogeneous catalyst ink. A 4 µL portion of the catalyst ink was transferred onto the polished glassy carbon electrode surface using a micropipette. The Pd loading on the surface of the glassy carbon electrode surface was 0.014 mg cm^−2^. Cyclic voltammograms were acquired in the potential range of −0.9 to 0.4 V with a sweep rate of 50 mV s^−1^, in 1 M KOH and 1 M KOH containing 1 M ethanol, respectively. Chronoamperometry was carried out at a potential of −0.3 V using 1 M KOH solution containing 1 M ethanol.

For the electrochemical CO-stripping measurements, CO was bubbled into the solution of 1 M KOH for 15 min after deoxygenation of the solution. N_2_ (99.9%) was then bubbled into the solution for 20 min to remove CO. During the process of ethanol electrooxidation in alkaline media in the presence of adequate OH^−^, the oxidation mechanism associated with CO_ads_ can be summarized as follows,

1 $${\text{Pd}} - {\text{CO}}_{\text{ads}} + 2{\text{OH}}^{ - } \to {\text{M}} + {\text{CO}}_{2} + {\text{H}}_{2} {\text{O}} + 2{\text{e}}^{ - }$$

All solutions were deoxygenated by bubbling with N_2_ before the tests, and all electrochemical measurements were carried out in a water bath at 25 ± 1 °C.

## Results and Discussion

**Fig. 1:**
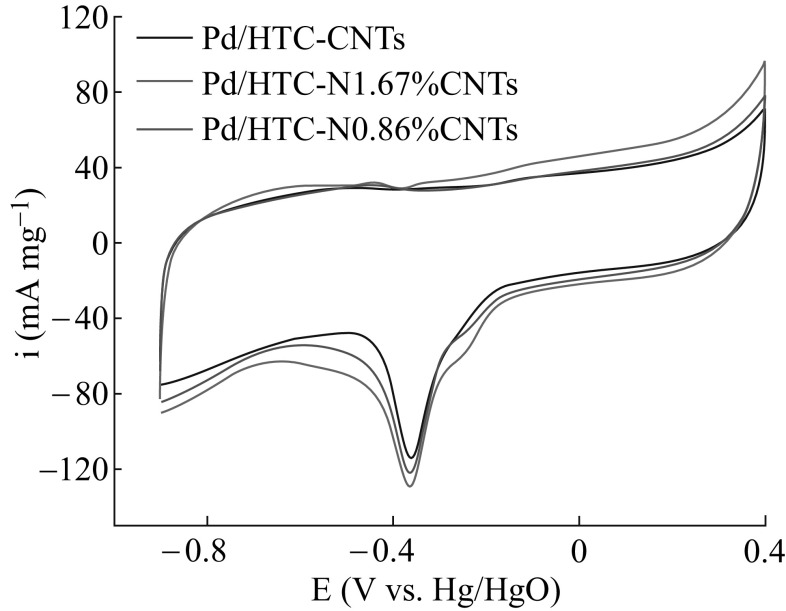
Cyclic voltammograms for different catalysts in 1 M KOH solution

The surface state of the three as-prepared catalysts was investigated by measuring the stable cyclic voltammograms in 1 M KOH solution (Fig. 1); the typical OH^−^ adsorption peak was observed in the scan from −0.6 to −0.4 V, and the reduction peak of PdO was observed in the reverse scan from −0.4 to 0 V [27, 28]. From comparison of these three catalysts, it is notable that the current density of the OH^−^ adsorption peak of the Pd/HTC-N1.67%CNTs was higher than that of Pd/HTC-CNT, where the current density of the former is useful for the removal of CO-like intermediates. More significantly, in the reverse scan, the reduction peak for the oxygenated species of the Pd/HTC-N1.67%CNTs occurred at much higher potential than that of the Pd/HTC-CNTs. The peak for the Pd/HTC-N1.67%CNTs also occurred at a lower potential, which is beneficial for ethanol oxidation.

**Fig. 2:**
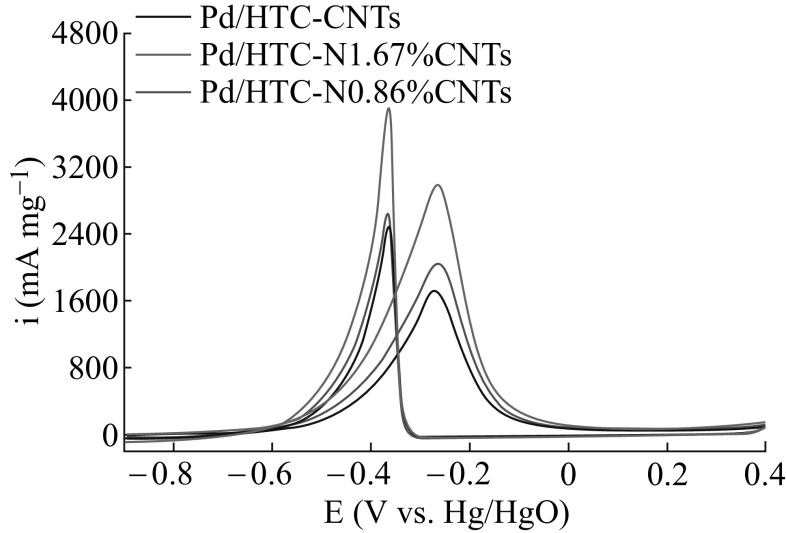
Cyclic voltammograms for different catalysts in 1 M KOH solution containing 1 M ethanol

Figure 2 shows the cyclic voltammograms for the three catalysts in 1 M KOH containing 1 M ethanol. The anodic peak observed in the forward scan corresponds to ethanol oxidation, which is of great significance for evaluating the activity of the catalysts [29]. The current density of the forward anodic peak of the Pd/HTC-N1.67%CNTs (2988 mA mg^−1^) was higher than that of the Pd/HTC-N0.86%CNTs (2052 mA mg^−1^) and the Pd/HTC-CNTs (1718 mA mg^−1^). This implies that the NCNT-supported Pd catalysts have higher catalytic activity for ethanol electrooxidation.

**Fig. 3:**
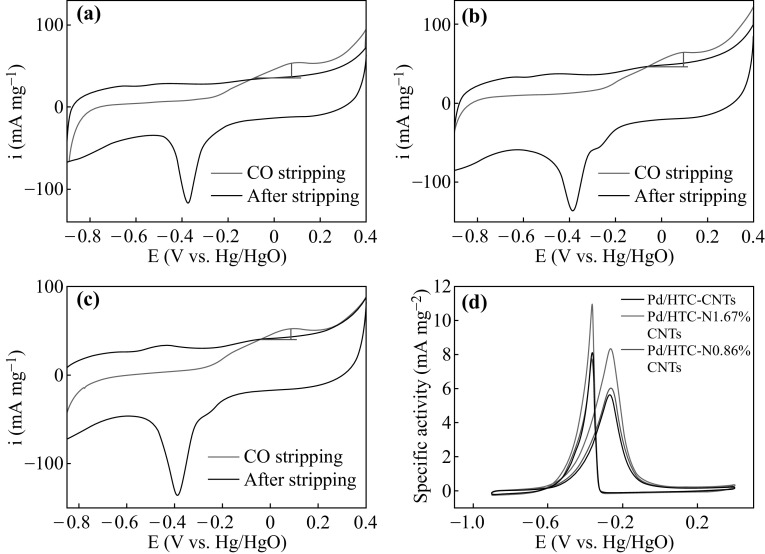
CO-stripping voltammograms for **a** Pd/HTC-CNTs, **b** Pd/HTC-N1.67%CNTs, and **c** Pd/HTC-N0.86%CNTs in 1 M KOH solution, **d** cyclic voltammograms of different catalysts in 1 M KOH containing 1 M ethanol (in Fig. 2), normalized by EAS

It is known that during the ethanol oxidation process, some reaction intermediates like CO will be strongly adsorbed on the Pd surface, resulting in poisoning of the catalyst. A CO-stripping test was conducted to evaluate the capability of the catalysts for the removal of adsorbed CO-like species. The CO-stripping curves of the three catalysts in 1 M KOH solution are shown in Fig. 3a–c. The electrochemically active surface area (EAS) can be estimated from the CO-stripping measurements. Typically, the CO-stripping curve can be determined by the electrochemical oxidation of pre-adsorbed saturated CO_ads_. The specific EAS of the catalyst can be calculated based on the following equation [30, 31],

2 $${\text{EAS}} = Q/mC$$

3 $$Q = 0.02\,S/m$$

where *Q* is the charge for CO desorption–electrooxidation, *m* is the Pd loading, and *C* (420 μC cm^−2^) indicates the conversion factor. The detailed calculation process is provided as follows:

According to Fig. 3a–c, the integral area (*S*) of Pd/HTC-CNTs, Pd/HTC-N1.67%CNTs, Pd/HTC-N0.86%CNTs are 6.43, 5.54, 7.17 mA mg^−1^ V, respectively. Based on Eq. 3, *Q* of Pd/HTC-CNTs, Pd/HTC-N1.67%CNTs, Pd/HTC-N0.86%CNTs are 12.86 × 10^−5^, 11.08 × 10^−5^ and 14.34 × 10^−5^ C, respectively. Based on Eq. 2, the EAS of Pd/HTC-CNTs, Pd/HTC-N1.67%CNTs, Pd/HTC-N0.86%CNTs is 30.6, 36.0, 34.2 m^2^ g^−1^, respectively. Finally, the calculated EAS values for the Pd/HTC-CNTs, Pd/HTC-N1.67%CNTs, and Pd/HTC-N0.86%CNTs were 30.6, 36.0, and 34.2 m^2^ g^−1^, respectively.

The cyclic voltammograms of the three catalysts were acquired in 1 M KOH containing 1 M ethanol (Fig. 2); the current normalized relative to the EAS is shown in Fig. 3d. The specific activity is often used to represent the intrinsic performance of catalysts. The anodic peak observed in the forward scan corresponds to ethanol oxidation, which is of great significance for evaluating the activity of the catalysts. It is clear that the forward anodic peak current density of the Pd/HTC-N1.67%CNTs (8.3 mA cm^−2^) is higher than that of the Pd/HTC-N0.86%CNTs (6.0 mA cm^−2^) and Pd/HTC-CNTs (5.6 mA cm^−2^). Chen et al. [32] reported that the specific activities of Pd/C and Pd/50CaSiO_3_/C were 2.7 and 4.4 mA cm^−2^, respectively. Therefore, the specific activity of the Pd/HTC-N1.67%CNTs catalyst in the present work is about three times higher than that of Pd-based catalysts in the literature.

**Fig. 4:**
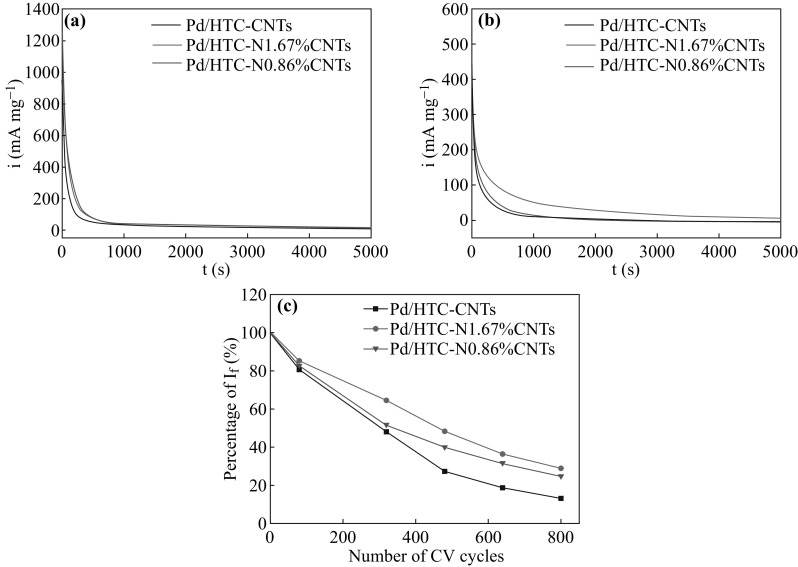
**a** Chronoamperometric curves for different catalysts, recorded at −0.3 V in 1 M KOH solution containing 1 M ethanol, **b** chronoamperometric curves for different catalysts, recorded at −0.25 V in 1 M KOH solution containing 1 M methanol, **c** peak current densities for different catalysts, recorded in 1 M KOH solution containing 1 M ethanol, as a function of the cycle number

To evaluate the stability of the catalysts, chronoamperometry curves were collected at the potential of −0.3 V in 1 M KOH containing 1 M ethanol (Fig. 4a). The Pd/HTC-N1.67%CNTs catalyst displayed the highest initial anodic oxidation current density due to its high electrocatalytic activity for ethanol oxidation, which corresponds to the activity results. Moreover, because of the formation of CO-like intermediates, the current densities of all the catalysts exhibited a rapid decline and reached a pseudo-steady state [33, 34]. Compared to the Pd/HTC-CNTs catalyst, the Pd/HTC-N1.67%CNTs catalyst exhibited a higher steady state current density with slower decay throughout the process of ethanol oxidation. However, to eliminate the influence of CH_3_COO^−^ during ethanol electrooxidation, as shown in Fig. 4b, another set of chronoamperometry curves were collected using 1 M KOH containing 1 M methanol, where CH_3_COO_ads_^−^ cannot be produced [28, 30]. From the evaluation of the performance of the catalysts toward methanol electrooxidation, it is verified that the Pd/HTC-N1.67%CNTs catalyst exhibits higher stability for the corresponding electrooxidation of ethanol. However, for comparison with the chronoamperometry curves, a more convincing and accurate test was carried out as shown in Fig. 4c. The cyclic voltammograms of all the catalysts were also measured in 1 M KOH solution containing 1 M ethanol for 800 cycles. After normalizing the peak current density of the catalysts as a function of the cycle numbers, it was clear that the Pd/HTC-N1.67%CNTs exhibited the highest peak current density for the process of ethanol oxidation. The remaining fraction of the peak current density increased from 13% for the Pd/HTC-CNTs to 24.8% for the Pd/HTC-N0.86%CNTs, and to 29% for the Pd/HTC-N1.67%CNTs after 800 cycles. Thus, the Pd/HTC-N1.67%CNTs catalyst demonstrated the best stability for ethanol oxidation, which is consistent with the chronoamperometry and CO-stripping voltammogram measurements.

**Fig. 5:**
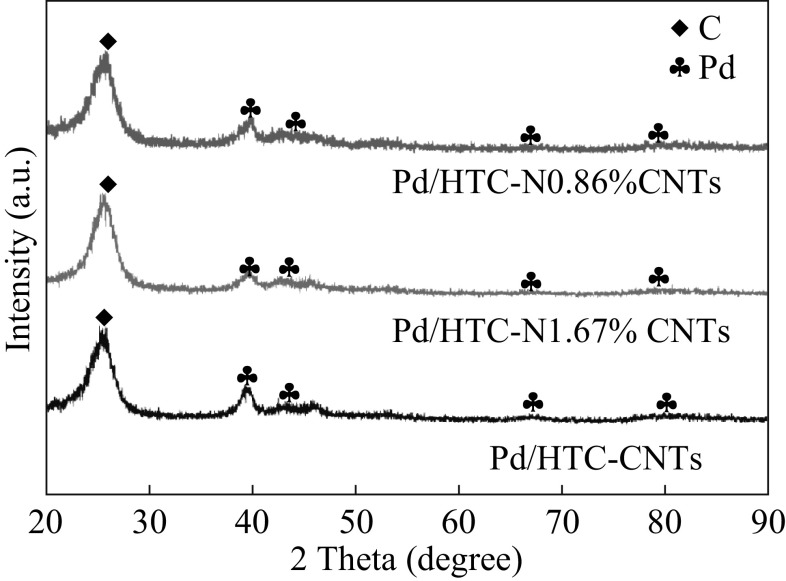
XRD patterns of different catalysts

Figure 5 shows the XRD patterns of the Pd/HTC-CNTs, Pd/HTC-N1.67%CNTs, and Pd/HTC-N0.86%CNTs samples. The diffraction patterns exhibit several peaks at 39.9° (111), 46.1° (200), and 67.5° (220), suggesting the formation of Pd nanoparticles. There was also a small amount of palladium oxide. In addition, the full-widths-at-half-maximum of these three catalysts were very close, which implies that the particle size of Pd was similar for all the catalysts.

**Fig. 6:**
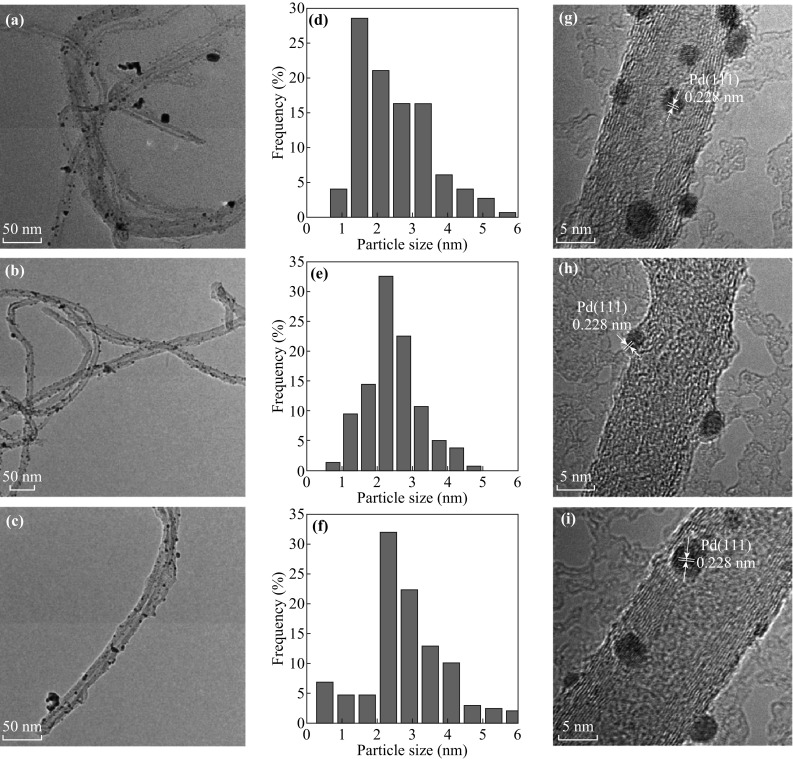
TEM images of **a** Pd/HTC-CNTs, **b** Pd/HTC-N1.67%CNTs, **c** Pd/HTC-N0.86%CNTs; **d**–**f** are the size distribution images for **a**–**c**, respectively. HRTEM images of **g** Pd/HTC-CNTs, **h** Pd/HTC-N1.67%CNTs, **i** Pd/HTC-N0.86%CNTs

Figure 6a–c, g–i shows the typical TEM and HRTEM images of the synthesized catalysts, respectively. It is clear that the Pd nanoparticles were uniform and monodisperse on all three surfaces. In addition, the average particle size of the Pd nanoparticles was about 2.5 nm for all the catalysts (Fig. 6d–f).

**Fig. 7:**
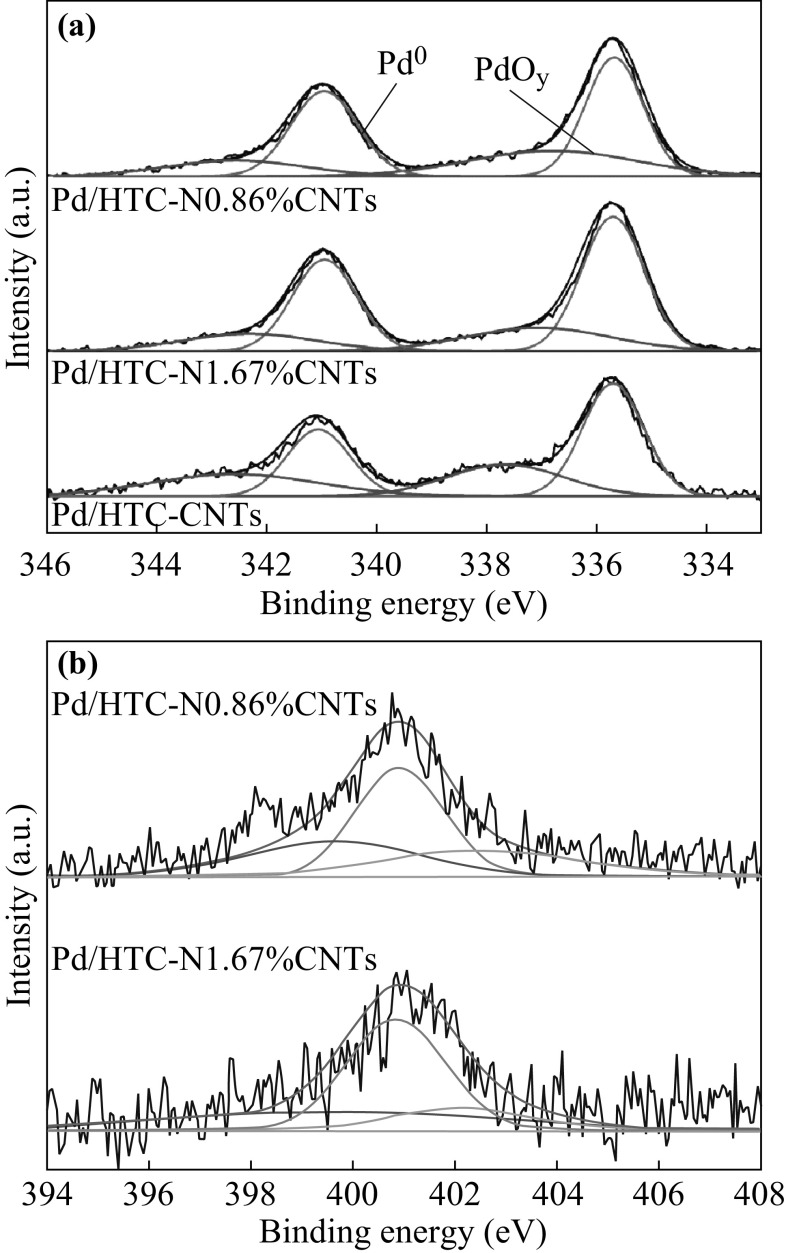
**a** XPS spectra of Pd/HTC-N0.86%CNTs, Pd/HTC-N1.67%CNTs, and Pd/HTC-CNTs in Pd 3d region, and **b** N1s spectra of Pd/HTC-N0.86%CNTs and Pd/HTC-N1.67%CNTs

The XRD and TEM data exclude the effects of the particle size and the dispersion of the Pd nanoparticles. Therefore, XPS measurements were conducted as shown in Fig. 7. The XPS Pd 3d spectra for the three catalysts (Fig. 7a) show peaks corresponding to Pd 3d_5/2_ (~335.7 eV) and Pd 3d_3/2_ (~341.0 eV) that can be ascribed to Pd and PdO_*y*_ (0 < *y* < 2) species, respectively [34, 35]. The respective Pd/PdO_*y*_ ratios were 1.5, 1.9, and 2.5 for the Pd/HTC-CNTs, Pd/HTC-N0.86%CNTs, and Pd/HTC-N1.67%CNTs. It is known that metallic Pd accounts for the electrocatalytic active sites in the catalyst and produces superior catalytic activity [22, 36]. Therefore, the highest fraction of metallic Pd in the Pd/HTC-N1.67%CNTs is consistent with its best electrochemical performance achieved with this system.

The Pd 3d_5/2_ peak was also negatively shifted for the Pd/HTC-N1.67%CNTs catalyst (340.95 eV) relative to that of the Pd/HTC-CNTs (341.05 eV) catalyst. Moreover, Fig. 7b shows a positive shift of the N1s binding energy from 400.75 eV for the Pd/HTC-N0.86%CNTs to 401 eV for the Pd/HTC-N1.67%CNTs. This confirms the interaction between Pd and N [37–39]. Therefore, due to the electron-donating effects of nitrogen, the electron cloud density of Pd may increase, which can stabilize Pd^0^, and the nitrogen groups impart a basic nature to the carbon surface and bind strongly to Pd, enhancing the Pd dispersion and preventing agglomeration of the Pd particles, thereby improving the electrochemical activity and stability of the Pd-based catalysts [21, 25, 34]. Accordingly, the Pd/HTC-N1.67%CNTs catalyst showed the best activity and stability toward ethanol electrooxidation.

**Fig. 8:**
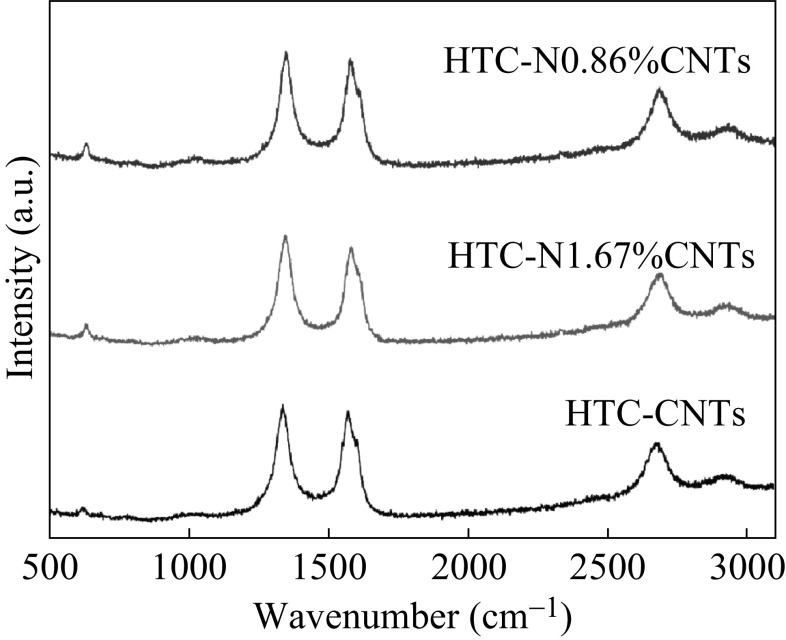
Raman spectra of HTC-N0.86%CNTs, HTC-N1.67%CNTs, and HTC-CNTs supports

The Raman spectra presented in Fig. 8 show the degree of graphitization of the CNTs. The profiles of all samples displayed three characteristic peaks at ~1343, ~1569, and ~2690 cm^−1^, assigned to the vibration of carbon atoms with dangling bonds and first-order scattering of the E2g phonons of the *sp* ^2^ carbon atoms, respectively. Narrow D and G bands accompanied by a strong 2D band indicate ordering of the graphite structure. Furthermore, the intensity ratio (*I*_*D*_ */I*_*G*_) of the three CNTs followed the order: 1.049(HTC-N1.67%CNTs) < 1.054(HTC-N0.8%CNTs) < 1.087(HTC-CNTs), indicating the presence of amorphous carbon layers with a number of defects after the HTC process. The defects on the surface of the NCNTs also help to anchor metallic Pd for ethanol electrooxidation.

**Fig. 9:**
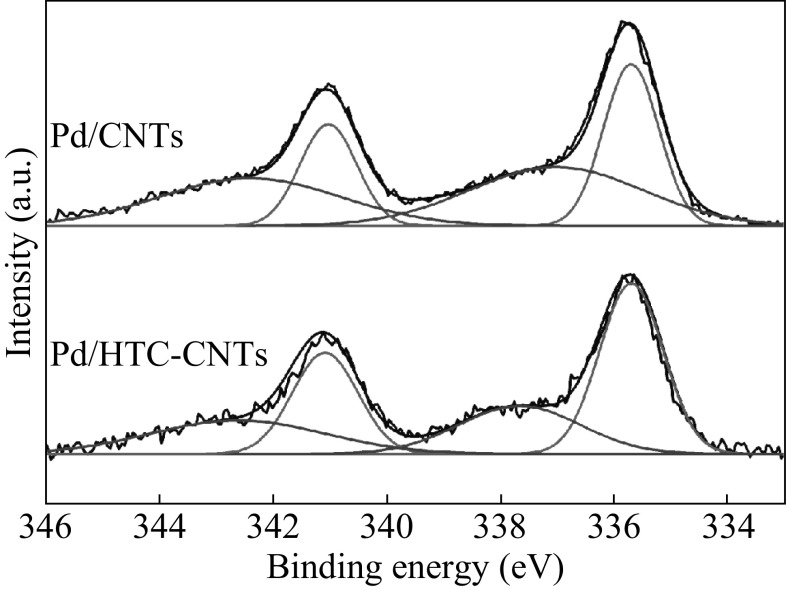
XPS spectra of Pd/CNTs and Pd/HTC-CNTs in Pd 3d region

The valence of Pd in the Pd/HTC-CNTs and Pd/CNTs catalysts was also determined by XPS analysis, as shown in Fig. 9. The Pd/PdO_*y*_ ratio was calculated to be 0.8 and 1.5 for the Pd/CNTs and Pd/HTC-CNTs, respectively. The results indicate that the defects introduced by the HTC process help to anchor metallic Pd for ethanol electrooxidation, which is beneficial for enhancing the catalyst performance.

## Conclusions

Three Pd-based catalysts supported on NCNTs with various nitrogen contents were synthesized via the HTC process. The Pd/HTC-N1.67%CNTs catalyst exhibited the best ethanol electrooxidation activity and stability as well as the highest specific activity. This improvement can be attributed to the interaction between nitrogen (doped in the CNTs) and the Pd nanoparticles, which favors the formation of metallic Pd in the catalysts. The HTC process also supplies a number of defects on the surface of the NCNTs that help to anchor metallic Pd for ethanol electrooxidation. The large proportion of metallic Pd not only facilitates OH^−^ adsorption and the removal of the CO-like intermediates, but also increases the specific activity of the catalyst, resulting in a significant improvement of the activity and stability of the Pd-containing catalysts.
